# Bibliographical Notices

**Published:** 1856-02

**Authors:** 


### BIBLIOGRAPHICAL NOTICES.

*Lehrbuch der Physiologischen Chemie.* Von Prof. Dr. C. G. Lehmann, *Zweite Auflage.* (*Zweite Umarbeitung*). Leipzig: Verlag von Wilhemn Englemann. 1853.

*Physiological Chemistry.* By Professor C. G. Lehmann. *Translated from the Second Edition*, by George E. Day, M. D., F. R. S., Fellow of the Royal College of Physicians and Professor of Medicine in the University of St. Andrews. London: Printed for the Cavendish Society, by Harrison & Son, St. Martin's Lane. 1851.

*Physiological Chemistry.* By Professor C. G. Lehmann. *Translated from the Second Edition*, by George E. Day, M. D., F. R. S., Fellow of the Royal College of Physicians and Professor of Medicine in the University of St. Andrew's. Edited by R. E. Rogers, M. D., Professor of Chemistry in the Medical Department of the University of Pennsylvania. *With Illustrations, selected from Funke's Atlas of Physiological Chemistry, and an Appendix of Plates.* Complete in two volumes. Philadelphia: Blanchard & Lea. 1855.

In the science of Physiology—yet so unsettled, and in which so few facts exist that are irrevocably established—we are dependent upon the personal views and opinions of those whom we recognise as the master minds among our teachers, to an extent that finds no parallel in any of the kindred sciences. Whilst in the study of the higher branches of Organic Chemistry, but few there are who may possess the talent required to foretel the result that was prophetically assumed by the experimentalist, yet when each new fact thus obtained is determined, it can be verified by the investigations of numbers who are fully competent to decide upon the truth or falsehood of the alleged discovery, for the conditions are now easily known, and alike within the reach of all. In the case of anatomy, and of all the allied simply descriptive sciences, the difficulty is still lessened, for here, each student can satisfy himself beyond all doubt. In physiology, however, and above all in physiological chemistry, how few can presume to form an opinion founded upon their own knowledge or research, or at the utmost do more than select one or two or more theories submitted to them by their respective authors, to whom they necessarily leave the advocacy of their individual merits. Not only is the opportunity for experiment upon the living organism restricted to the few, who may possess a thorough knowledge of chemistry in all its branches, united with anatomical and surgical skill, but of these experiments thus performed by those who are most competent, and upon whose labors every reliance must be placed, how often do we find the results of to-day contradictory to those of yesterday, and that which we believed finally settled by the investigations of one physiologist, shown by another, upon further research, to be error and delusion. The varied influences of health and disease, are not under our control like those of heat and cold. The phenomena of digestion, respiration, or the circulation of the blood, do not admit of being represented by a simple arithmetical equation, like those of solution, diffusion or the osmotic force. The processes of vitality are too obscure to permit us, in many cases, to expect any other than a negative or indecisive result, and if, after repeated experiments and repeated failures, a definite solution is at last obtained, we can never feel perfectly satisfied that every contingency has been provided for, and every source of error removed. Under these circumstances, therefore, the physiological chemist cannot draw his inferences, nor establish his theories with the almost mathematical precision now attained to in the other branches of the science.

Besides these difficulties, it is absolutely necessary that the physiologist should be able to view his own labors in their true light, and not through the distorted medium of partiality, that too easily leads all men, but those possessing the soundest common sense as well as genius, to ignore or undervalue the labors of others when placed in competition with their own. It is no wonder, therefore, that but few obtain to an elevated position in this science, since the requisites are talent of the highest order, incessant and unwearied industry, a cultivated mind, well versed in all the kindred sciences, and above all a clear and unbiassed judgment.

It is but seldom that we have perused the works of any author wherein the possession of the above essentials is so fully proved as in the writings of C. G. Lehmann. No reader of the work now under our notice can help perceiving the moderate and calm though firm and independent manner in which he maintains his own opinions, and reviews those of others. The true value of the theories, with the facts upon which they rest, are here given us, and everything is done that lies in the author's power, to enable us to judge how much, or rather how little we know of the world and life within us.

The first endeavor of the author is to give in the methodological introduction, an outline of the difficulties connected with this study, owing to the false notions entertained of the applications and capacities of chemistry, and of the erroneous statements that have been made, and still are made by those ignorant of the science. Deductions are too often drawn from carelessly performed experiments, and arranged in a purely imaginative manner, in the form of chemical equations and formulæ, with no foundation in truth whatever. Attempts are made to judge of the metamorphoses of the blood, &c., grounded solely upon an examination of some few of its constituents, and executed in a manner violating all the rules of science. Substances are often stated to have their probable origin in the disruptions of certain other compounds, whose very existence in the organism is without proof, and often probability. In this manner, the semblance of exact investigation is carried out, and the fiction of chemical symbols made the cloak of ignorance. Another frequent source of error, is the utter absence of all details regarding the condition of the patient during disease, where analysis has been made of morbid products, the name of the disease being all that is usually given, which of course is no guide to the physiological chemist. Too much, also, is expected from chemistry in proportion to the present advances of the science. Animal chemistry is still unacquainted with a correct and practically useful method of analysing the blood; we do not know, even by name, many of its constituents. Fibrin cannot yet be exhibited in a chemically pure state. We know not how the globulin of the blood corpuscles is secreted; we cannot separate the so-called protein oxides, and much more in the blood is still but imperfectly known. We analyze healthy and morbid milk, and yet are ignorant of what substances the mixture we call *casein* is composed of.

The great importance of chemistry, in the attainment of any correct knowledge of the processes of vitality, is dwelt upon at length by the author. To use his own language, "It cannot be denied that most of the phenomena of animal life consist in, or are accompanied by, chemical processes, nor can we form an adequate conception of the functions of the nervous system, by which sensuous motion and perception are regulated without the simultaneous existence of chemical action." He considers that it occupies the highest place among the sciences auxiliary to medicine. The futility of drawing a distinction between physiological and pathological chemistry is forcibly illustrated, together with the folly of wasting time upon the examination of morbid fluids or tissues, when we are still ignorant of the nature and composition of the same in their normal state. Pathological phenomena do not originate in new laws or special forces, but simply from an alteration in the surrounding conditions. When iron is no longer precipitated by an alkali, owing to the presence of tartaric acid, or fibrin ceases to coagulate when certain salts are present, these substances do not become *abnormal;* they are still governed by the same laws as in other states.

The physician may call certain inflammatory phenomena abnormal and morbid, but the philosopher perceives only the necessary results of laws, acting under different relations, for *he* has to deal with fixed laws, and not with rules abounding in exceptions, "for there is but one physiology, as there is but one all powerful law of nature."

No attempt is made by the author, to arrange the compounds he treats of in a physiological classification, either as nutrient or excretory matters, since we often find a substance apparently belonging to the latter class, in one part of the body, applied in another part to the formation of tissue or other important function. Any divisions into secreted and excreted matters, are only the means of endless confusion.

The expressions progressive and retrogressive metamorphosis of matter, are only useful so far as they serve as the means of generalizing certain facts; as a strictly scientific designation applied to particular processes, they are without value, since no leading principle is embodied in their meaning, for it is generally utterly impossible to decide when or where the progressive formation ceases, and the retrogressive commences.

Our limits will not permit us to give a detailed account of the several chapters devoted to the organic substrata. Among the latest views and experiments, the following occur:—*Oxalic acid* in small amount is almost always found in the form of oxalate of lime, in normal human urine, particularly when it has been allowed to stand for some time; a portion thus found may be formed by the decomposition of uric acid, after it leaves the system, into oxalic acid, allantoin and urea; it is satisfactorily proved, however, that it exists under circumstances precluding the possibility of this change. From his experiments, he finds it most frequently in diseases affecting the respiratory processes, and when the pulmonary tissue has lost its elasticity from repeated catarrh, but not so often in tuberculous or inflammatory affections of the lungs. It is common in convalescence from severe diseases, as typhus, &c. His experiments do not corroborate Golding Bird's statement of its occurrence in dyspeptic patients.

It is the opinion of the author that *butyric acid* is one of the products of the destructive metamorphosis, being probably one of the products of the oxidation of the nitrogenous or fatty matters. Farinaceous matters are sometimes converted into butyric acid in the primæ viæ, but the belief that this is the first step towards the formation of fats, he considers utterly without support of probability.

Liebig's view of the occurrence of *benzoic acid* in the urine of horses, when hardly treated and poorly fed, was not corroborated by the author's experiments, who found hippuric acid *in all cases* where the urine was fresh, but detected benzoic acid as soon as fermentation had commenced. Benzoic acid is one of the few acids producing a decided acidity in the urine.

The salts of *lactic acid* obtained from animal fluids differ from those obtained from the fermentation of sugar in their amount of water of crystallization, solubility and decomposition by heat. The magnesian and zinc salts, however, were found by Lehmann to be alike from both sources. Lactic acid has become of great interest lately, from the beautiful discovery of Strecker, showing that in all probability it consists of formic acid coupled with aldehyde. Since alanine, which consists of aldehyde and prussic acid is converted by nitrous acid into nitrogen, water and lactic acid, and further, since the decomposition of lactate of copper supports the view that aldehyde pre-exists in lactic acid, this theory may be considered as established. Lehmann has lately prepared the lactates from a larger quantity of gastric juice than had heretofore been obtainable; determined their atomic weight, and proved the acid of the gastric juice to be identical with lactic acid. Hydrochloric acid is also found therein with both free lactic acid and lactates. From the experiments of Schmidt, compared with his own, he believes that the lactic acid in the gastric juice is sometimes entirely replaced by hydrochloric acid, under conditions that were undeterminable by him. The author is firmly convinced of the occurrence of the lactates in normal blood, although direct proof thereof can probably never be given, owing to their rapid oxidation into carbonates, and their removal by other means. They are proved to exist in chyle and muscular fibre, and are again found in the urine, consequently must have passed through the blood. Scherer believes that they exist in large amount in the blood of puerperal fever, the blood having an acid reaction. Lehmann has only thrice observed an acid reaction of the blood; once in pyæmia in a man, and twice in women shortly after delivery.

Lactic acid does not exist in normal milk, excepting upon a strictly animal diet, when milk having an acid reaction is secreted. Its existence in the muscles is now firmly established. Liebig, who positively denied its occurrence there, now think it exists in sufficient quantity to saturate all the alkaline fluids of the body. Although Lehmann formerly earnestly maintained that the acid reaction of the urine depended upon its presence, he is now satisfied that it is only an occasional constituent thereof. Whenever oxalate of lime is abnormally present in the urine, lactic acid is always associated with it. With reference to the origin of lactic acid, Lehmann attributes it to two sources; from amylaceous food in the primæ viæ, but principally to the metamorphoses of muscular fibre and other tissue. The only objection to this latter view rests upon the fact that this acid has not been as yet in any manner produced out of the body, from either albuminous or gelatinous bodies; this, however, is not a fatal objection, since we cannot expect to produce the same metamorphoses in all cases in our laboratory that nature effects in the organism. Its production from nitrogenous matters, viewed in a theoretical light, is quite possible. The alkaline lactates undergo rapid combustion in the blood, forming carbonates, and may be considered the best supporters of animal heat known.

Among the basic nitrogenous bodies, *creatine* is considered (not only from physiological grounds, but also from Liebig's investigations,) simply as an excretory matter like urea. Its similarity, both in constitution and chemical relation, to caffein, gives it no title to be ranked among the nutrient substances, since the latter can be viewed only as a stimulant, incapable of contributing to the formation of tissue.

In his experiment on *urea*, the author found that the nature of the food exerted a great influence on the amount contained in the urine. The increase of urea occurs very shortly after the food is taken, five-sixths of the nitrogen being often eliminated within twenty-four hours, by the kidneys in the form of urea. Lehmann rejects the possible existence of a so-called urea diathesis, since the increased excretion of urea is not the cause, but only an uncertain consequence of certain diseases. He corroborates the existence of urea in normal blood, as stated by Simon, Lieberkuhn, and others. The amount is, however, extremely small, not accumulating in an hour above .021 per cent. of the weight of the blood. Urea is primarily formed in the blood, and not in the muscular fluids, tissues or kidneys, a large portion of it probably arising from the decomposition of uric acid.

Strecker has recently succeeded in preparing *taurine* artificially from the isethionate of ammonia, by heating it to 210$^0$ C. thus adding another to the list of substances, that like urea, were considered at first as being obtainable through the medium of vitality alone, but which now are readily produced by the chemist at will.*

The appearance in the urine of uric acid in the form of urate of soda (which was mistaken by Golding Bird for urate of ammonia) is not to be regarded as a pathological symptom, since its appearance is produced by simple physiological causes, want of exercise or any other cause preventing the blood properly penetrating into the pulmonary vessels. In emphysema, heart disease, enlargement of the liver, etc., as well as in fevers, where the proper relation between the circulation and respiration is no longer maintained, this amorphous impalpable yellow sediment (Prout) is often met with. His own experience contradicts Bird's view, that there is an increased secretion of uric acid in gout; before the paroxysm, and in chronic gout, (when chalky deposites are found) the amount is always diminished in the urine; whilst in rheumatism, particularly in acute articular, its amount in the urine is very much increased. When *free* uric acid is found in the urine, it is always a symptom of some severe morbid process. Lehmann regrets that the conception of gout, as entertained by physicians, is so vague that no meaning scarcely can be attached to the term. No pathological distinction has as yet been given distinguishing gout from rheumatism, their assertions to the contrary notwithstanding; and the group of symptoms considered characteristic of the latter has been proved by ana-

* Isethionic acid is produced by the action of anhydrous sulphuric acid upon olefiant gas. The common coal gas answers the purpose very well. It has the formula $C_4\ H_5\ S_2\ O_7$ .—REVIEWER.

tomical observation to be deceptive. The diseased condition of the osseous system, the earthy deposits and concretions, may all exist independently of gout. No distinctive mark exists, excepting the deposits of urate of soda, but these seldom occur, and the disease must have made great progress before the malady can be diagnosed by their presence. The pretended oxidation of the constituents of the blood, which was thought to explain phthisis, gout and stone, is not the simple process by which specific diseases can be explained scientifically. "There are no acute and but few chronic diseases in which the oxidation of the constituents of the blood is not impeded or diminished, and there is *no* disease characterized by too sudden or rapid oxidation of the blood." The increase of uric acid in fevers, etc., is most probably produced by the retardation of the oxidation of uric acid into urea and oxalic acid. Uric acid stands in Lehmann's opinion higher in the scale of metamorphosis than urea does.

As regards the settlement of the long dispute concerning the generation of *fat*, whether it was derived from the other constituents of the food, as maintained by Leibig, or whether it was simply contained in the food taken by the animal, Leibig's view has been fully proved to be correct. Lehmann does not support Leibig and Scherer's view that the primæ viæ are the seat of the formation of fat from vegetable food, since the results of the experiments of Tiedemann and Gmelin, Boussingault, H. Meckel, and others, give no sufficiently definite reason to form this conclusion. Besides the evidently mechanical and physical uses of fat in the organism, such as its supporting and protecting the various organs, its non-couducting power, etc., the author thinks that it plays a far more important part in the animal economy than is generally ascribed to it. Without being disposed to view it so much as a stored up supply of nourishment for the body, intended, as Leibig thinks, principally for fuel for combustion, and therefore easily deposited and taken up again, which the cellular structure of its containing vessels with their albuminous walls, etc., render improbable, he considers that it is entitled to the most prominent place among the constituents of the body, as one of the most active agents in the metamorphosis of matter. He has found that a small portion of fat is absolutely necessary to the metamorphosis and solution of nitrogenous food in the process of digestion. An examination of the intestinal villi during digestion, and a comparison of the relative amounts and character of the fat in the chyle contained in the finest lacteals, with the contents of the thoracic duct, he thinks, is sufficient to prove the important influence it exerts upon the metamorphosis of the albuminous constituents of the nutrient fluid. Its invariable occurrence under all circumstances in the blood, and its making an essential part of the separate nerve fibres, so that after the extraction of fat they remain as hollow cylinders only, renders it highly probable that it is indispensable to the functions of the nervous system. The investigations of Achinson, Hünefeldt, Nasse, and others, tend to prove that fat is the predisposing cause of cellular formation. The newly secreted plasma always contains more fat than after the nuclei or cells have been deposited, and all plastic exudations contain more fat than nonplastic do, although the latter often contain much cholestrin, yet but little true fat. It is to this disposal of fat that the beneficial use of cod liver oil is to be attributed, whose utility, he thinks, is now beyond dispute; to consider it as merely a supply of fuel for respiration when the lungs are to a great extent blocked up and useless, is in his opinion absurd. Whenever the progress of cell formation shall be represented by an equation, fat will form one of the integral factors thereof, since it in all probability co-operates in the metamorphic action, and itself experiences metamorphoses, combinations and decompositions. Its participation in the formation of bile is already almost beyond doubt, cholic acid being in all likelihood a conjugated fatty acid, the presence of oleic acid in it being determined although not yet isolated. Many other physiological reasons render this view nearly irrefutable.

The formation of *Glucose* (or sugar) in the system, from starch, etc., is effected beyond all doubt by the pancreatic juice. The fact of its formation from nitrogenous substances is now decided, by the experiments of Bernard, Frerichs and himself* (see 3d edit.) who have determined its seat of formation to be in the liver. The view of Bernard, that the formation of sugar in the liver is increased by irritating the base of the fourth ven-

* It is not so stated in the Cavendish or American editions.

tricle he considers erroneous, since to support it, it would be required to be shown that the rapidity of circulation of the blood through the liver was increased, and that other secondary products should also be found, which experiment has not done. Many other physiological causes are also opposed to this view. In his opinion, the increased amount of sugar and the acidity of the urine in diabetes, after the operation above mentioned, are produced by the retardation of the oxidation of the sugar that normally exists in the blood, causing it to accumulate therein, this effect being dependent upon the disturbance of other forces in the system, and not upon the action of the liver. Besides the observation of Schrœder, that the roots of the vagus were *not* touched in this operation, Von Becker has found that injury to several other parts of the brain produce the same effect, although the medulla oblongata was not disturbed. His own experiments have shown that after injection of grape sugar into the veins of a rabbit, it voids, within ten or fifteen minutes, a still alkaline urine, but containing sugar ; at a later period its urine still contains sugar but the alkalinity has diminished; at the end of from five to seven hours the urine becomes acid and contains but little if any sugar ; in nine hours the urine has generally become again alkaline and not a trace of sugar could be detected. It is probably owing to these conditions, that Bernard has fallen into the error of asserting that grape sugar does not pass into the urine when injected into the veins, since he must have delayed the examination for sugar, when a large quantity was injected, to eight hours or upwards, or from three to four hours when a smaller quantity was made use of. Stannius found that the sugar is rapidly removed (within twenty-four hours) from the bladder even where no urine has been passed.

The formation of *Hæmatin* in a *chemical* point of view is as much of an enigma as ever ; from physiological analogies there is reason to believe that the hæmatin is first formed within the perfected cell, that the granular matter found within the blood corpuscles is fat, which was the origin of the colorless blood corpuscles and first recognizable within the minute lacteals. Weber and Kölliker have demonstrated that blood corpuscles are found in the liver in the fœtal state, and during the hibernation of certain animals. The examination of the development of the chick within the egg, justifies the assumption that fat takes part in the formation of Hæmatin. This view he offers only as an hypothesis since the data are too few to depend upon with confidence. The uses of Hæmatin are still undecided, since we do not know what relation the artificially prepared substance bears to the original condition in which it existed in the blood. The labors of Virchow render it very probable that it is ultimately metamorphosed into melanin or bile pigment.

The division of nitrogenous substances into the so-called *Protein* bodies is retained by Lehmann merely for the convenience it affords in facilitating examinations, and not for the sake of the theory therein embodied. The difficulty attending the study of these bodies, from the want of any means of distinguishing the separate members of the family from each other, gives to most of the experiments connected therewith only a relative certainty, especially in the case of coagulated albumen and the other allied substances, when met with in a solid state; it is evident, therefore, how absurd it is to attempt to decide whether it is albumen or fibrin that exists in tubercles or carcinoma, although many pathologists think they have settled this point without the aid of chemistry.

With reference to the disputed point whether *Fibrin* separates in threads or lamellæ, the author states that no doubt can exist in the case of inflammatory blood where the corpuscles sink rapidly, and the fibrin coagulates slowly, when the off-shooting of individual threads and their gradual augmentation in various directions can be clearly seen. Whether these filaments increase subsequently in breadth as well as length, and thus give rise to lamellæ, no definite answer can as yet be given. Microscopical examination, as well as numerous physiological arguments tend-to show that fibrin is not a simple homogeneous substance, the analysis thereof can have at most an approximative value only. After reviewing the occurrence and properties of fibrin, the author arrives at the conclusion that it is derived from albumen, and not from the protein—containing food. The only hypothesis he thinks of any value, concerning its probable origin is, that it is produced by the oxidation of albumen; and although at first sight somewhat of a paradox, that the increase of fibrin in inflammatory fevers, pneumonia, etc., is due to a

*diminution in the supply of oxygen*, the frequent though short and imperfect respirations serving to convey sufficient oxygen into the blood to convert it into fibrin, but not sufficient to oxidize it further; it therefore attains its maximum in pneumonia and pleuritis, the blood becoming rich in carbonic acid, since this gas is excreted only in proportion to the oxygen absorbed. The physiological importance of fibrin lends force to this argument.

According to Lehmann, the position occupied by fibrin is intermediate between the so-called progressive and retrogressive metamorphosis; he considers it as a product of oxidation of albumen, alike fitted to be oxidized either into new tissue or into the excreta, as the wants of the organism may require, or the amount of albuminous substance received in the food exceed the demands of the system. When disease prevents the proper renovation of tissue it accumulates in the blood until the excreting organs remove it. No difficulty need here be found if it is not attempted to force nature into the artificial divisions of progressive and retrogressive metamorphosis.

We have already mentioned that Lehmann regards fat as one of the most essential substances in the formation of *bile;* another element associated therewith is sugar, which he is disposed to consider conjugates with oleic acid, losing six equivalents of water and forming cholic acid. He agrees with Frerichs in the view that the sugar found in the parenchyma of the liver contributes with other substances to the formation of bile; a large portion of the sugar, however that is formed within the liver is carried into the blood through the hepatic veins. All that is known with any precision regarding the function of the bile, may be thus stated. Besides the nitrogenous and non-nitrogenous substances conveyed by the portal vein, most of which are in a state of metamorphosis, substances also pass into the liver which must be regarded as secondary products of the process that gives rise to the formation of blood cells; the fats and certain mineral substances particularly belong to the latter class, and fibrin and hæmatin are the most prominent among the former. Hence the bile may be viewed, not as the product of the metamorphosis of any one morphological or chemical constituents of the body, but as an assemblage of compounds formed by substances chemically distinct that meet in the liver, in a state of metamorphosis, whence their elements unite in the nascent condition giving rise to the mixture called bile. The participation of bile in the digestion of fat has been definitely settled by the experiments of Bidder and Schmidt, although a small quantity of fat can be absorbed when the access of bile to the intestine is prevented. The only explanation that can be given of this fact is that bile in some manner modifies the relations of adhesion between the oily mixture and the moist animal membrane, which direct experiment has proved, although the rationale is unknown.

The experiments of Frerichs and of Bidder and Schmidt, totally contradict and disprove Bernard's assertion of the co-operation of the *pancreatic juice* in the digestion of fat. The error that he fell into, appears to have been dependent upon the length of time that elapsed after the operation in the animal, before the observation on the action of the intestine was made. The digestion of amylaceous food is, however, effected by this fluid, conjointly with the saliva, the latter exerting a subordinate action only.

A dispute has existed for some time, between Lehmann and Bence Jones, regarding the conversion of ammonia in the organism into nitric acid—the latter maintaining its oxidation—the former denying it, principally upon the ground of insufficiency in the method of testing; he also thought ammonia very rarely occurred in normal urine.*

The experiments of Henle and of A. Nasse prove that the relative color of the *blood* is dependent upon the form of the the corpuscle, for when blood is diluted with water, the fluid becomes much darker in color, and on examination each globule is found to have altered in form by endosmosis, becoming biconvex instead of concave, thus refracting and dispersing the rays of light; the investing walls of the corpuscle become also thinner, permitting the dark color of the contents of the glo-

* Neubauer has lately decided by his experiments that Lehmann's views regarding the non-oxidation of ammonia were correct, but that it does exist in normal urine to the amount of one-tenth of one per cent. When muriate and other salts of ammonia were administered they passed unchanged in their full amount into the urine.—*Jour. für prak. Chem. Mai* 1855. Reviewer.

bule to be seen more perfectly through the membrane. Solutions of neutral salts, sugar, &c., produce the opposite effect. Harless and Nasse have determined that oxygen and carbonic acid also alter the form of the corpuscle and consequently its color. Harless, in his experiments upon frog's blood, found that oxygen made the outline of the corpuscle very dark, the cell walls very finely granular, the nucleus of an oval form, but not very distinct, and the contents of a pale yellow color. Carbonic acid increased their dimensions, made their form almost spherical, the capsule perfectly transparent, the nucleus distinct and with a clear outline, and the contents redder than in the previous experiment. No conclusion can be drawn from the jagged or torn appearance which the blood globule presents in some diseases; it is known that the presence of common salt in moderate amount will produce this appearance, explaining perhaps why the portal blood frequently contains such and not the hepatic blood, since the former contains much more salt than the latter. The alternate action of oxygen and carbonic acid upon the blood, besides darkening the color, also acts chemically upon the walls of the corpuscle. Harless found that after ten such alternations, the globule was destroyed. Nasse found that no deductions could be drawn from experiments upon the action of chemical agents out of the organism, with reference to their probable effect when administered to the living animal, since the results are often the very reverse of what might have been expected. When solutions of bicarbonated alkalies were injected into the jugular veins of horses, it affected them with all the symptoms of intoxication by carbonic acid; other experiments prove that the bicarbonate is converted in the blood into neutral carbonate and carbonic acid. Lehmann considers that there can be no doubt whatever that the carbonic acid in the blood exists to a great extent in a state of chemical combination, although so slight that many causes, such as exposure in a vaccuum, passing neutral gases through it, &c., suffice to decompose it, since the same effect is produced by the same agents upon a solution of bicarbonate of soda (or potash), in which form the carbonic acid probably exists in the blood. Liebig thinks that the same conditions apply to oxygen, which Lehmann considers, as probably though not positively true, since the blood is capable of absorbing fourteen times as much oxygen as the same amount of water would. This capacity for condensing or combining oxygen is principally vested in the corpuscles, and most likely in the hæmatin, since it has this property, when isolated, in a high degree. The particular duty performed by the blood cells, is still to a very great extent a matter of conjecture; all that can be stated, with any amount of probability, regarding its functions, is that it is an organ standing in a definite and intimate relation with the plasma in which it swims, and serving as a laboratory in which the various constituents of the plasma are fitted for the ultimate purpose of assisting in the formation and renovation of tissue.

Lehmann adopts Vierordt's view of the method by which the interchange of gases takes place within the lungs, founded upon the laws of absorption discovered by Dalton and Henry. The latter has shown that the amount of gas absorbed by a fluid is dependent upon the pressure exerted by that gas upon the surface of the fluid, and that the presence of another gas in no way affects this presence, one gas diffusing into the atmosphere of a second as into a vacuum. If, therefore, there is a greater amount of carbonic acid in the blood than the presence of the carbonic acid in the pulmonary vesicles is able to keep in a state of condensation, then the excess will escape into the atmosphere through the delicate membrane with which it is in contact, until the amount be reduced proportionate to the pressure. A motion in the opposite direction will take place with the oxygen, since the blood on entering the lungs contains much less oxygen than it can condense at that pressure, the gases therefore interchange until these conditions are satisfied.

We have already exceeded the limits assigned to us, and yet have scarcely glanced at a few of the most prominent among the many admirable observations of the author. Our extracts have principally been confined to the first half of the work, since the more elaborate and detailed statements of the various processes of digestion, respiration, nutrition, the structure of nervous tissue, etc., would require for their elucidation in even the most concise form possible, far more space than is at our disposal. We must not, however, omit directing the reader's attention to the chapter on vital forces, which we think cannot but prove highly interesting to all, from the beauty of the style as well as the importance of the subject.

The *first* German edition of this most admirable work appeared in 1841. In the beginning of 1850, the first volume of the *second* edition appeared, and at intervals during the space of three years, the other volumes were published. The progress made by the science during the lapse of nearly ten years had been so great, that the whole subject required rewriting—thus forming an entirely new book. The demand for this edition was so great that it was exhausted almost immediately after its completion—again affording the author an opportunity of revising it, making such alterations and additions as the advanced state of physiological chemistry required, and by the simultaneous publication of the three volumes preventing the unavoidable contradictions and errors that must occur in every scientific work of so progressive a nature as this, where three years are allowed to expire between the appearance of the first and last volumes. The *third* edition, although differing from the preceding one, far more than is usually the case, by one of those nice distinctions that are only to be found among the Germans, still retains the title of second edition, though the words second remodelling* are added to distinguish it from the preceding edition. Throughout its text, references are made to Otto Funke's well known "Atlas of Physiological Chemistry," whenever these exquisite plates could lend assistance to the student.

In 1851 the Cavendish Society published the first volume of Dr. George E. Day's translation of Lehmann's work, and in 1853 and 1854 the remaining volumes were issued. This translation being of the second edition, of course contained the errors appertaining to the original. In the last volume, therefore, the translator gave an appendix consisting of notes and additions, the additions being taken from Lehmann's third and latest edition, desiring, no doubt, to render the translation a correct representation of the author's opinions and statements at that date. But, unfortunately this, as we will subsequently show, was but imperfectly done, leaving many errors and contradictions, particularly in the first volume, and thus detracting from the value of the work. Dr. Day's reputation as an accurate and competent translator is so well established, that it is scarcely necessary to

*Zweite Auflage. Zweite Umarbeitung.

remark upon the fidelity of the translation or the perspicuity of the language. In a work of this size, however, it is of course impossible to avoid all errors. We have noticed a few where the meaning is directly the reverse of that given in the German text; in some instances the error is not unimportant; thus, in describing the importance of taking into account the general condition of the system, when an analysis is made of a morbid exudation, the translator says, "There is nothing of chemical pedantry in desiring parallel analyses of the blood but yet the accuracy of a physico-scientific inquiry would not be invalidated by its omission." The true meaning of the passage is, "the desire for a parallel investigation of the blood is anything but pedantry, although even the *latter* could not invalidate the accuracy of a scientific investigation,"* this error renders the whole passage obscure. An error of a similar nature occurs in page 307, 2d vol. Amer. edition, where the meaning conveyed is directly the reverse of that intended. The American is an exact copy of the Cavendish Society's edition, with the single exception that the notes and additions contained in the appendix to the latter are distributed throughout the text where they properly belong. A number of wood cuts taken from Funke's Atlas also occur at intervals through the work, and a number of plates from various pathological and anatomical notes are annexed to the end of the book.

When we consider the eminent position occupied by this work, when we remember that its author is now the highest authority upon the science which he has done so much to advance; when we find his name occupying the most prominent position in every physiological work we chance to open, and when his latest views or discoveries substantiate or overthrow the theories of all who have gone before him, we find ourselves utterly unable to understand why this reprint of the Cavendish Society's edition, copied with all its errors and deficiencies should have been given us, when a subsequent German edition, not translated however, has been published nearly three years. Apart from the frequent and important alterations in the method of arrange-

* Das verlangen paralleler Blutunter suchungen ist aber nichts weniger als Pedanterie, wiewohl diese der Genauigkeit, einer naturwissenschaftlichen Untersuchung nie Eintrag thun wird. Bd. 3. S. 128.

ment, we find many statements occurring in the American work that subsequent investigation have altered, and which are corrected in the third German edition. In comparing the first volumes of the German and American editions, we were obliged, on almost every page, to notice some greater or lesser discrepancies that existed between them; in some cases several pages are found entirely new in the German edition. We need but instance that in the translation the *bare possibility* of the formation of sugar from nitrogenous substances is noticed; whilst in the 3d edition, the German, it is stated as being beyond doubt established that sugar is furnished from nitrogenous substances, probably fibrin, and that the seat of its formation is the liver; several pages containing the author's views thereon being entirely rewritten. The American edition barely mentions, as needing further confirmation, the experiments of Bernard upon the increase of sugar in the urine by irritating the base of the fourth ventricle; the third edition gives the views (such as are given in outline in the preceding pages) and the deductions therefrom with experiments founded upon them. In the translation, the old view of the constitution of stearic acid is given, and a theory explanatory of its formation from margaric acid. In the German edition the discovery of its being isomorphous with margaric acid is mentioned; the former theory of its mode of formation is therefore omitted. In the translation the substitution theory is given in outline only, the author being evidently undecided which to adopt in his explanations. In his later work, the theory receives full elucidation and Kolbe's numerous discoveries are used with the confidence they merit. We might thus increase the list to any extent if needed; we commenced writing down the variations between the two editions until we found them too numerous to make use of.

From the high position and reputation possessed by Prof. Rogers we cannot but think it must have been the particular wish of the publishers that the work has appeared in its present condition, since we feel confident that no gentleman in our country possesses in a higher degree both the ability and disposition to have brought the work up to the present state of the science. We regret also that we cannot consider the wood-cuts interspersed through the book, and at the end thereof as an addition to the value of the work. When the low price that Otto Funke's Atlas can be obtained for is taken into view, we think it would have been much better to have followed the plan pursued by the author in his last edition, of making reference to the Atlas throughout the text. How far the plates in the American edition are valuable from their accuracy may be judged of by the fact, that in the plate copied from Funke's Atlas illustrating the coagulation of human blood, the artist has entirely *omitted the fibrin* which is so beautifully shown in the original plate and which is the *essentially characteristic feature* of the drawing.

Publishers in this country of works of a high scientific value, like the one before us, appear to form a very inadequate idea of the purpose to be fulfilled by the plates chosen for illustration. They are not intended to satisfy an idle curiosity, but to serve as an assistant as well as teacher to the student, enabling him to recognize the morphological elements in the microscopic field when first presented to his view. It is upon this fidelity to nature far more than upon their exquisite artistical execution that the great value of Otto Funke's Atlas depends.

---

*Transactions of the American Medical Association.* Vol. viii. 1855.

*Report on Deformities after Fractures.* By Frank Hastings Hamilton, M. D.

There is no surgical subject in which the profession at large is more interested than that of fractures.

The frequency with which they occur and the serious nature of the consequences following them, render any practical investigations connected with the subject, interesting to a large majority of the profession. These injuries cannot be disposed of by a country medical practitioner, on the ground that he is no surgeon; he cannot *send them to a city*, and thus get rid of the responsibility and the trouble; but is obliged, from the nature of the case or from the impossibility of moving the patient, to treat them at home, and oftentimes to rely upon his own ingenuity for mechanical contrivances, with the application of which, heretofore, he may have had little experience. In a large city, a poor man man can be sent to a hospital, or in the case of one whose means are more fortunate, where a consultation can be afforded, he can bring to his aid those skilled in surgical practice ; but in the country, the refusal to treat such an injury, or a want of proper success in the attempt, would be disastrous to the business and to the reputation of the physician ; therefore, we may say, there is no paper in the volume before us, that will be read with greater interest, than that of Prof. Hamilton upon deformities after fractures.

The unsightly deformities so frequently seen, the unfortunate cripples with useless limbs which meet us on every side, and the law-suits, disgraceful to all parties concerned, which we constantly hear of, render the subject, also, one of no ordinary interest, one fraught, in fact, with paramount importance to the community as well as the profession.

To be able to state to a patient with the positiveness of law, and without the fear of subsequent contradiction by results, that the fracture in his case must be attended with precisely so much or so little shortening or deformity, is the highest aim of surgical prognosis. Every patient and every physician should have a clear understanding of what is to be expected; there should be no guess work ; we should be able to say, you can be cured without deformity, or on the other hand, you must necessarily have such deformity and such inconvenience, as inherently belongs to the case. The American Association have shown their appreciation of the importance of the subject, by appointing a commission upon it, and placing it in the hands of one who has entered upon the prosecution of his labors with the greatest zeal and assiduity.

Various and confused are the ideas on the subject of deformities, in some measure dependent upon medical education and training, want of familiarity with the literature of the subject, or personal experience in the matter. The great difficulty at the outset of an undertaking like that upon which Dr. Hamilton is engaged, is the determining of a standard or an average, or rather the difficulty which meets us at the onset is to *define deformity* after fracture. Are we to receive these words literally, that is, are we to say that deformity has resulted, if upon boiling a bone we find there has been some perceptible though slight deviation from normal length and form, although during life there had been no inconvenience in the use of the part or perceptible disfigurement, or are we to consider the term as implying perceptible disfigurement during life, and some limitation to the use of the limb ? This is the first question to be settled at the onset of such an investigation. Is deformity to be determined osteologically, or by deviation from normal functions and conformation ? In the next place, how much deformity is to be expected ? Into the settlement of this question, a great many elements enter, and therefore it is a subject upon which no hasty conclusions are to be drawn. We must have extensive fields of observation ; the materials of our conclusions require a long time for their collection, and the statistics which are to be of value in determining our conclusions, must be collected with great care ; we must know how the limb was treated and who treated it, and how it was measured before it can form an element in our calculations, and then again even after having determined the average amount of shortening and deformity in any bone, we must be careful how we apply this knowledge in forming our judgment of any particular case ; we are not to say this is a good or bad cure unless we know all the circumstances, for in some instances, the patient may have occasion to congratulate himself, even if his shortening and deformity exceed the average, and on the contrary, another might have occasion to complain, should his fall short of the average. We mean here to intimate, that it will be difficult to express in general terms, by measurement in inches, the degree of shortening which is to be anticipated in a fracture. So much depends upon the age of the individual, the density of bone, the force producing the injury, and the direction of it, the character of the tissues, particularly the muscular, the susceptibilities of the patient, his power of endurance, and his capacity for reparation ; there are so many variable elements in fine, exclusive of the skill of the practitioner, which may influence the result, that it is hard to lay down any law in inches for deformity after fractures.

In the third place, in determining in our own minds what is avoidable or unavoidable as the result of fracture, we shall experience great difficulties arising from different degrees of manipulative skill in equally well informed practitioners; in basing conclusions upon statistical reports, therefore, much must depend upon the confidence we have in the ability of the surgeon who has had charge of the case.

In making such a report as this, we fully concur with Dr. Hamilton that it is not only important to avoid "the error of encouraging the practitioner with a prognosis too favorable," but it is equally important to avoid that of "leaving him to expect too little."

Dr. Hamilton certainly enters upon this investigation with the idea that deformities after fractures are much more common than is generally admitted, and implies that they have not been published sufficiently, and that the teachings and writings of the present day would encourage the expectation of more favorable results than can possibly be obtained. He states that there has been an unwillingness upon the part of his professional brethren to reveal their want of success, and says:—

"It is certain that, up to this moment, no one has volunteered to state fully what have been the results in his own practice, or in the practice of the hospital, or other similar institutions, which have been under his immediate charge. In hospital records, you may find patients admitted with fractures, and reported as 'dead,' or as dismissed 'cured,' with the occasional interpolation of 'a good leg;' and, upon these records, tables have been constructed to determine the average fatality of such accidents, and the probabilities of cure; but I have not yet seen any published reports declaring what was the exact amount and value of the 'cure'—how much the bone was shortened, or bent, or otherwise maimed and deformed. In short, they still fail to inform us what are the 'deformities after fractures,' which, under fair treatment, may reasonably be expected."

After this, Dr. H. cites statistics of fractures occurring in several hospitals in this country and abroad, and complains that they are "*silent upon the* subject of deformities or shortening," "and that the same disinclination to approach the subject is manifested by those who have written special or general treatises upon fractures," with a few exceptions, and that "in looking for an explanation of this seeming indifference, or palpable ignorance," he finds "several probable causes." The first of these is an inability to recognise the shortening from a want of a proper mode of measurement. Secondly, that hospital surgeons dare not "record faithfully their results in the treatment of fractures," because the eyes of their pupils, their governors, their confréres, and of the public are upon them."

"To be honest in the admission of shortcomings in a branch of our art, where the difficulties are so little understood by those who constitute themselves our judges, would be suicidal. Do you not see that jealous and designing colleagues would have no such discreditable results? Pupils would draw unfavorable comparisons, and desert the wards where failures were so common; rival hospitals would secretly or openly seize upon such records to their own advantage; and the patients themselves would, no doubt, often return the diligence, skill, and honesty of their surgeons with imprecations and prosecutions."

And he avers also, that "there is no lack of charity in these suppositions."

Thirdly, a defective education of students on the subject.

"Students will continue to go out from our hospitals with a belief that perfect union of broken bones is the rule, and that exceptions imply generally unskilful management; and if, when hereafter they have themselves occasion to treat a fractured femur, the result falls short of their standard of perfect success, they, taught also by the same instinct of self-preservation which actuated their teachers, will conceal the truth from others, and even from themselves, if possible. Nay, I fear that sometimes, under the same urgent promptings, and where the moral sense is not superior to all other considerations, they may hesitate to regard the sanctity of an oath!"

We are free to admit, that there is a great deal of truth in these charges, although they may seem severe. There certainly is but one true mode of measuring a fractured limb. There are, certainly unworthy men in high and responsible positions, and there is a great deal of erroneous surgical teaching. We can heartily join with Dr. Hamilton in rebuking these errors, so far as they exist. We may express our regret with him, that the subject of fractures is so much neglected, that so many think they understand it, when they really do not; that it is so unattractive to students, that we have even heard of their requesting their professor to abandon the subject for another more agreeable to them; we can also wish that he had alluded in this connection to the present system of cultivating a taste in students for bloody surgery at college clinics, in preference to the witnessing in hospitals of this important class of injuries; we wish he had added, that a student who only attends a college clinic, and depends on this for his clinical instruction, can never be competent to treat a serious fracture, for who ever saw a fracture of the thigh, or a recent compound fracture of the leg at a college clinic? We could have wished, however, that he had made some exceptions in his general charge; as we feel that such exceptions are due to some, whose lessons in the wards we have valued, and by whose instructions we have profited; we think there are surgeons who do know how to measure limbs, and who are not afraid of jealous rivals or public opinion, and who honestly teach medical students the unfavorable as well as the favorable results. We should like to defend some institutions and some teachers from the imputation, but Dr. Hamilton will admit of no such defence, unless we can accompany it "with proofs of the same specific character and obtained in the same careful manner," as those which he presents in his report. Nevertheless, we are unwilling to allow the Pennsylvania Hospital, of this city, to remain under this charge, because we cannot furnish Dr. Hamilton with published statements concerning the points at issue; for we can appeal with safety to the personal knowledge of those who have attended its teachings, if not to the properly kept statistics, that there has been no desire to conceal deformities after fractures; that there has been but one mode of measuring fractured limbs; that there are some high-minded surgeons above the fear of pupils, governors and confréres; and that, *there*, students have been properly taught the treatment of fractures. We feel it a duty to rescue from this charge those to whom we have alluded, although they have neither written nor published their teachings in such a way as to enable us to quote from them; nevertheless, we have no doubt that those connected with this institution would be willing to bear censure, provided that the great object that Dr. Hamilton has in view in writing this paper can be effected, at although we feel perfectly certain they would have no reason to complain if there could be presented to the world all the results of their treatment.

The first chapter in the Report contains a history of 22 cases of fractures of the Ossa Nasi, only 13 of which were seen by a surgeon in time to afford relief, and of which only 3 are tabulated as perfect cures.

"Of the thirteen cases to which a surgeon was called at first, three died within a short time, and no attempt to restore the ossa nasi was deemed necessary or proper. In two other cases it is probable that the nature of the injury was not recognized. Of the eight remaining cases, and in which attempts at restoration were immediately made by qualified surgeons, five are marked imperfect."

Dr. Hamilton's remarks, with reference to the nature of this injury, its liability to be neglected, and the mode in which the deformity is to be removed, are eminently practical, and exhibit an experience which entitles them to consideration; he shows, also, by experiments upon the dead subject, that the cribriform plate of the ethmoid is not so frequently broken or disturbed in connection with this fracture, as is usually taught.

Chapter second contains a history of 7 cases of fracture or displacement of the Septum Narium, all of which are tabulated as imperfect. In his remarks upon this subject he says:—

"Of the 'results of treatment' in cases of fractures or displacements of the septum narium, we have no facts or conclusions to record. In three of the seven cases no surgeon was employed, and in these, also, no surgical treatment was adopted, and it is quite probable that in only a small proportion of all the cases was the nature of the accident recognized."

Chapter third contains 6 cases of fractures of the Ossa Maxillaria Superiora, one of which is reported as perfect. In connection with this injury, Dr. H. suggests a procedure for elevating the malar bone, in cases where it has been driven firmly into the antrim:—

"I wish then to suggest a procedure, the value of which I have not yet had an opportunity to determine by any experiment upon the living subject, but which I have carefully and frequently tested upon the dead. It is only that we should employ for the purpose of lifting the malar bone, a screw levator, an instrument which I find already constructed, in a case of trephining instruments made for me by Mr. Luer, of Paris, and which I have often used and constantly recommended to my pupils, in certain cases of fractures of the skull. The instrument ought to be made of the best steel, and with a broad, sharp-cutting thread. A slight incision being made through the skin, and down to the centre of the malar bone, the elevator is then screwed firmly into its structure, and now its elevation and adjustment may be accomplished with the greatest ease. In case the surgeon is not provided with this excellent instrument, a joiner's gimlet might answer tolerably well."

Chapter fourth contains an account of 18 cases of fractures of the Maxilla Inferior. Dr. Hamilton says:—

"The amount of deformity resulting, also, from these fractures is usually very trifling, whatever treatment has been adopted. Seven are marked imperfect, but one of these cases was complicated with other injuries of which the patient died in a few days, and one was a case of delayed union, at the time of writing this report. Only five of the united fractures are imperfect, and in none of these is the imperfection such as to be noticed in a casual examination of the face. The deformity which is usually found, is a slight irregularity of the teeth, produced, in most cases, by a falling of the anterior fragment, and in one case by a slight elevation of the anterior fragment. But even this does not always interfere with mastication, and would often pass unnoticed by the patient himself. It is probable, too, that time, and the constant use of the lower jaw in mastication, will gradually effect a marked improvement in the ability to bring the opposing teeth into contact. I think I have observed this in several instances."

In connection with this subject, he suggests the use of gutta percha, to be moulded when soft, upon the teeth, thus keeping the parts in their proper position; he has also given a figure of an apparatus for fracture of the lower jaw, composed of leather straps, of which he says:—

"The advantage of this dressing over any which I have yet seen, consists in its capability to lift the anterior fragment almost vertically, and at the same time it is in no danger of falling forwards and downwards upon the forehead."

Chapter fifth contains 53 cases of fractures of the Clavicle. The frequency with which this accident occurs, and the generally acknowledged difficulty in maintaining the fragments in their proper position, will impel the reader to look to this portion of the report with more than ordinary attention. Dr. H. divides these fractures into two classes, complete and incomplete, and assigns to the latter 14 cases, and the former 39. To us, this seems a large proportion of partial fractures. The occurrence of fourteen incomplete fractures in fifty-three cases, makes the ratio greater than 1 in 4. We have no doubt about the diagnosis, but should hesitate to draw any conclusion as to the frequency of this particular injury from so limited a table, and it is hardly probable, that in the next fifty-three cases this kind of injury would be so frequent.

Dr. H. commences his remarks upon these cases with some observations upon the occurrence of incomplete or partial fractures, the existence of which has been denied by many eminent surgeons—but concerning which, there can be little doubt at the present time. The complete fractures are classified according as the accident occurs in the inner, outer, or middle third, as well as into simple and comminuted. From these cases, Dr. H.

shows where the fracture is most likely to occur, and also points out the relative position of the fragments.

The *prognosis* is the point of particular interest, however.

"Of 39 complete fractures, only seven have united without shortening; and of 25 simple, oblique, complete fractures, which have occurred at or near the outer end of the middle third, only one has united without any shortening (case 45); and in this case the patient was but fifteen years old, and the fragments were never much displaced, nor can I say that the treatment had anything to do with the result. Three cases of complete *transverse* fracture, occurring at the same point, have united without the shortening."

"In one instance, a transverse fracture, the union seemed to be tolerably firm at seven days."

He quotes expressions from many surgical writers, Syme, Miller, Fergusson, Velpeau, Vidal (de Cassis), Desault, Physick, as evidence of the difficulty in treating this fracture without deformity; he then passes in review the different modes of treatment which have been adopted in various times by different surgeons, and finally says: "no apparatus perhaps, has been so generally employed among American surgeons, as that form of the sling introduced by Dr. George Fox, at the Pennsylvania Hospital in 1828." He says:—

I believe, also, that, in some measure, this general preference is due fairly to the intrinsic excellences of the dressing. But I must be permitted to express a doubt whether it has made deformities of the clavicle 'the exception, instead of the rule,' with us. I have used this dressing, as may be seen by a reference to my cases, oftener than any other form, and yet my success has by no means been so flattering as has been the success of these gentlemen. I have seen others employ it, also, and with pretty much the same results. Nor ought it to be forgotten that, in Great Britain, by far the greater majority of surgeons employ an apparatus essentially the same. I have seen it in many of the hospitals, and Mr. Bickersteth, one of the Surgeons of the Liverpool Infirmary, informed me, in 1844, that it had been in use with them as long as thirty years."

In another paragraph he says:—

"If it is intended, however, to say that a shortening is not generally prevented, but only that no unseemly projection of the fractured ends will be found to result, I reply, that then it does not answer *all* the indications, and I beg, further, to suggest that the avoidance of an upward projection seems, to me, to depend much more upon that part of any apparatus which lifts the shoulder, and which belongs to a multitude of other forms of dressing as well as to that in question, than upon that which forces the shoulder out, and it may be accomplished, in a majority of cases, as well without an axillary pad, with a mere sling, as with it. But in fact, my experience has convinced me that the absence or presence of such a projection, after union, is due more to the circumstances of the fracture, as to whether it is more or less oblique: and still more especially, to the degree of roundness, or emaciation of the patient, than to any form, or part, or condition of the apparatus. It will be found more distinct in oblique fractures than in transverse, and much more marked in thin persons than in plump, or fat persons, and more so in muscular than in non-muscular. In short, I affirm that such a projection has occurred as often under my observation, when this dressing has been used, as it has when other forms have been employed.

Finally, while I deprecate incautious assumptions in regard to the capabilities of any form of dressing for broken collar-bones, a disposition to which is manifested by more than one advocate of special plans, I am ready to bear my humble testimony in favor of that one of whose claims I have taken the liberty to speak so freely, and which is usually known in this country by the name of Fox's apparatus, consisting essentially of a sling, axillary pad, and bandages to secure the arm to the chest, and to which the stuffed collar is a convenient accessory, but admits of various modifications, answering the same ends. Among the considerable variety of dressings which I have used, this, either with or without such slight modifications as I shall presently suggest, has seemed to be most simple in its construction, the most comfortable to the patient, the least liable to derangement (if I except Velpeau's dextrine bandage), and as capable as any other of answering the several indications proposed."

Dr. Hamilton suggests improvements upon Fox's apparatus, and its application, recommending a smaller pad, and the maintaining of the elbow in a line with the body; in the appendix there is a wood-cut of the author's apparatus, together with others that have been invented for a similar purpose.

We have given our readers as fair a summary as lies in our ability of Dr. Hamilton's valuable paper, though we regret that our limits have not allowed us to extract more largely from it. It affords us the warmest pleasure to state, that his paper has been prepared with a great deal of care, and exhibits not only great practical knowledge upon the subject which he treats, but an earnestness and desire for truth which must commend itself to every candid reader. We feel confident that it will be productive of much good; that it will call attention to an important subject, and bring before the mind of every one who undertakes to treat a fracture, how much shortening is justifiable in the case or must necessarily result.

We would, however, caution those who might, perhaps, take comfort to themselves from this report, and out of these cases of "deformities after fractures," justify their bungling and negligence.

Dr. Hamilton, we need hardly say, is not responsible for any such perversion of his paper. We know that he is the last man who would give "aid and comfort" to pretending ignorance, as he certainly does not intend to be quoted hereafter by those who have produced some most horrible deformities, as countenancing the idea that cures *can never* be made without deformity.

Finally, we congratulate Dr. Hamilton upon the results of his labors, and shall look forward to his next report before the American Medical Association with high expectations.

*Prize Essay—Statistics of Placenta Prævia.* By JAMES D. TRASK, M. D.

The profession must ever take a deep interest in all that relates to cases of placenta prævia, as it is justly considered one of the most serious complications incident to parturition. Liable at any moment to be called to the aid of a patient, whose life and that of her child depends on his prompt and decisive interference, every practitioner must find the necessity of, and entertain a lively sense of gratitude to those whose researches have furnished him with, settled rules for his guidance on such embarrassing occasions.

Since the days of Guillemeau, until Dr. Rigby, of Norwich, introduced a more discriminating practice, turning was always resorted to in cases of alarming hæmorrhage before delivery. The latter gentleman, in his essay, drew the important distinction between partial and complete implantation of the placenta over the os uteri, and showed that in instances of the former, rupture of the membranes and other temporising measures, generally sufficed to bring the labor to a safe termination, while in cases of the latter, he maintained that turning afforded the patient the best chance of recovery. On this basis the practice of the profession uniformly rested, until Dr. Simpson, of Edinburgh, announced the somewhat startling doctrine, that in certain cases, the extraction of the placenta alone was preferable to turning, asserting that "in 19 out of 20 cases in which it has happened, the attendant hæmorrhage has been either altogether arrested, or it has become so much diminished as not to be afterwards alarming."

It may be stated in general terms, that the cases in which Dr. Simpson recommends the extraction of the placenta, are in all instances where flooding is urgent and uncontrollable, the os uteri being at the same time undilatable; and again in cases of alarming prostration, in which turning would be extremely dangerous, yet the hæmorrhage continuing unchecked.

The "new practice" has many able advocates, but is warmly opposed by Drs. Lee, Ashwell, &c., and to the laudable desire of contributing to the elucidation of this *vexata questio* we are indebted for the very able essay of Dr. Trask.

The essay gives a statistical history of 353 cases of complete or partial placental presentation, arranged in three separate tables. The first table gives the result of 251 instances in which *turning* was resorted to, the second shows the success in 36 instances of *spontaneous separation* and *expulsion* of the placenta before the birth of the child; the third the result of 66 cases where the placenta was *artificially* separated and *extracted.*

We would gladly transfer to our pages the interesting summaries of these tables, but our space will not permit. Therefore, after stating that Dr. Trask's laborious researches fully substantiate the soundness of Dr. Simpson's recommendation, we must content ourselves with assuring our readers, that there is no question relating to placenta prævia, that is not satisfactorily elucidated in this admirable essay.

---

*The Practitioner's Pharmacopœia and Universal Formulary: containing* 2000 *Classified Prescriptions, selected from the practice of the most eminent British and Foreign Medical authorities. With an abstract of the three British Pharmacopœias, &c.* By JOHN FOOTE, M. R. C. S., London, &c. With corrections and additions by an American Physician. New York: S. S. & W. Wood, 261 Pearl Street, New York.

Though we believe that books of Prescriptions frequently exert a very injurious effect, we must acknowledge that such works, judiciously employed, are often of assistance to the practitioner.

Dr. Foote's formulary is an excellent one of its class, and we take pleasure in commending it to the profession.

---

*The Anatomical Remembrancer, or complete Pocket Anatomist, &c., 2d American from the 4th London Edition. With corrections and additions.* By C. E. Isaacs, M. D., Demonstrator of Anatomy in the University of New York. New York: S. S. & W. Wood. 1855.

This little work is well calculated for the purpose it is intended for, which is to recall to the mind of the student the information previously acquired, as well as to assist him while engaged in the details of Practical Anatomy.

The corrections and additions of Dr. Isaacs, the American editor, add much to its value.

---

*Pronouncing Medical Lexicon, containing the correct pronunciation and definition of most of the terms used by speakers and writers on medicine and the kindred sciences. With additions.* By C. H. Cleveland, M. D. &c. Cincinnati: Longley Brothers. 1855.

We have compared this work with Dr. Reese's Medical Lexicon, of which it is accused of being a copy, and find that many of its definitions are the same. It has, however, numerous technical terms in it, not to be found in Dr. Reese's work. The pronunciation of each word will be readily understood by those familiar with the Phonotypic Alphabet, in which they are all given, and which constitutes the peculiarity and main excellence of the work. No references are made to the derivation of the words contained in the work.

---

*Synopsis of the Course of Lectures on Materia Medica and Pharmacy, delivered in the University of Pennsylvania.* By Joseph Carson, M. D. Philadelphia: Blanchard & Lea. 1855.

Prof. Carson's views of the necessity and advantages of the above "frame-work," must commend themselves to every student who is anxious for aid in obtaining and securing the instructions orally given him.

"To the character of an independent treatise the work presents no claim; in fact a large proportion of it requires the explanations given in the lecture-room."

Though particularly dedicated to the students of the University of Pennsylvania, it cannot fail to be equally serviceable to students in other Institutions.

